# Case report: Targeted sequencing facilitates the diagnosis and management of rare multifocal pure ground-glass opacities with intrapulmonary metastasis

**DOI:** 10.3389/fonc.2023.1276095

**Published:** 2024-01-23

**Authors:** Yingshun Yang, Guotian Pei, Mingwei Li, Xiaoxue Ma, Shuai Wang, Xianjun Min, Shushi Meng, Jiayue Qin, Huina Wang, Jun Liu, Yuqing Huang

**Affiliations:** ^1^ Department of Thoracic Surgery, Beijing Haidian Hospital (Haidian Section of Peking University Third Hospital), Beijing, China; ^2^ Department of Medicine, Acornmed Biotechnology Co., Ltd, Beijing, China; ^3^ Department of Thoracic Surgery, Aerospace 731 Hospital, Beijing, China

**Keywords:** multiple primary lung cancer, intrapulmonary metastasis, thoracoscopic surgery, EGFR tyrosine kinase inhibitors, circulating tumor DNA, case report

## Abstract

**Introduction:**

Treatments for multiple ground-glass opacities (GGOs) for which the detection rate is increasing are still controversial. Next-generation sequencing (NGS) may provide additional key evidence for differential diagnosis or optimal therapeutic schedules.

**Case presentation:**

We first reported a rare case in which more than 100 bilateral pulmonary GGOs (91.7% of the GGOs were pure GGOs) were diagnosed as both multiple primary lung cancer and intrapulmonary metastasis. We performed NGS with an 808-gene panel to assess both somatic and germline alterations in tissues and plasma. The patient (male) underwent three successive surgeries and received osimertinib adjuvant therapy due to signs of metastasis and multiple *EGFR*-mutated tumors. The patient had multiple pure GGOs, and eight tumors of four pathological subtypes were evaluated for the clonal relationship. Metastasis, including pure GGOs and atypical adenomatous hyperplasia, was found between two pairs of tumors. Circulating tumor DNA (ctDNA) monitoring of disease status may impact clinical decision-making.

**Conclusions:**

Surgery combined with targeted therapies remains a reasonable alternative strategy for treating patients with multifocal GGOs, and NGS is valuable for facilitating diagnostic workup and adjuvant therapy with targeted drugs through tissue and disease monitoring via ctDNA.

## Introduction

Recently, the detection rate of lung cancer in patients with multiple ground-glass opacities (GGOs) has increased (1–4). GGOs are generally considered multiple primary lung cancers (MPLCs), but some cases of intrapulmonary metastases (IMs) with GGOs have been reported (2, 5). Effective differentiation between MPLC and IM is directly related to subsequent management. In difficult cases, exploring the clonal relationship of multiple tumors may help to distinguish synchronous MPLC (SMPLC) from IM (6–10). For SMPLC, surgical resection of suspicious malignant lesions should be the first choice when it is medically and technically feasible (3, 11). However, the management of residual GGO lesions is often challenging for SMPLC patients who do not have the main tumor resected at the same time, and close postoperative surveillance is essential. *EGFR*-tyrosine kinase inhibitors (TKIs) can provide a noninvasive treatment for unresectable GGOs in SMPLCs with *EGFR* mutations (12). However, the treatment of multiple GGOs with both MPLC and IMs will be more complicated.

Plasma circulating tumor DNA (ctDNA) consists of DNA fragments in the blood that contain tumor-specific somatic alterations. The blood sample can be collected with minimal discomfort occurring. Serial analysis of ctDNA has been shown to track tumor burden (13, 14) and to correlate with treatment-driven clonal evolution (15–18). Recent advances in the detection of ctDNA have shown promise in monitoring minimal residual disease (MRD) by tracking ultralow-frequency somatic tumor mutations and monitoring recurrence in patients with nonmetastatic cancer, including non-small cell lung cancer (NSCLC) (19–21). Likewise, ctDNA can be used to circumvent the problem of spatial heterogeneity (22). However, ctDNA detection in multifocal lung cancers has rarely been reported. Herein, we describe a patient with rare lung cancer who presented with more than 100 GGOs, was diagnosed with both MPLC and IM, and was subjected treatment of multiple complex GGOs.

## Case presentation

**Figure 1:**
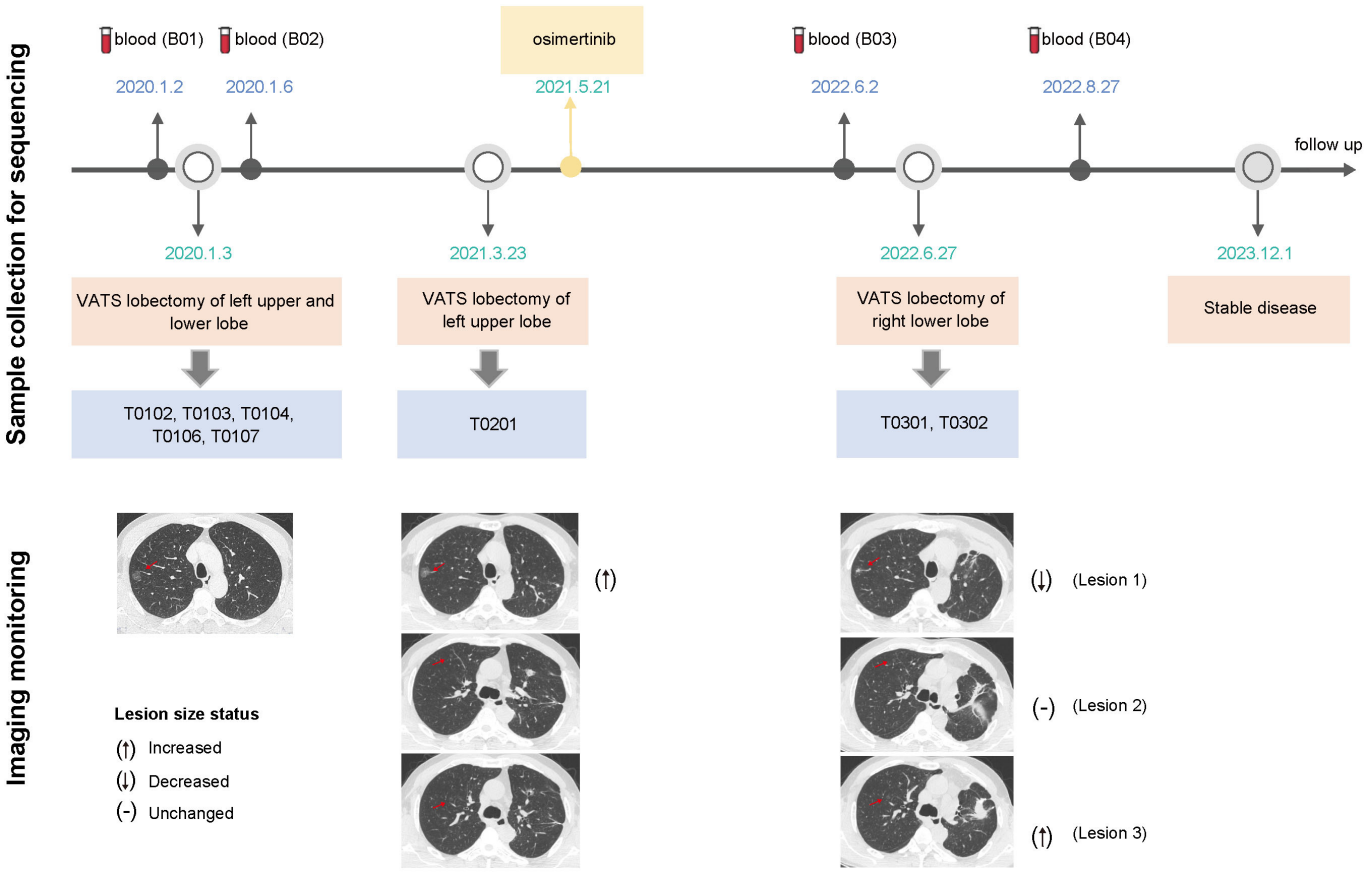
Timeline describing the clinical course, samples collected for sequencing and treatments administered (upper). Imaging assessments were performed using computed tomography scans to track treatment efficacy (lower); the CT images revealed three scenarios in which the lesion size changed before and after osimertinib administration; VATS, video-assisted thoracoscopic surgery.

**Figure 2:**
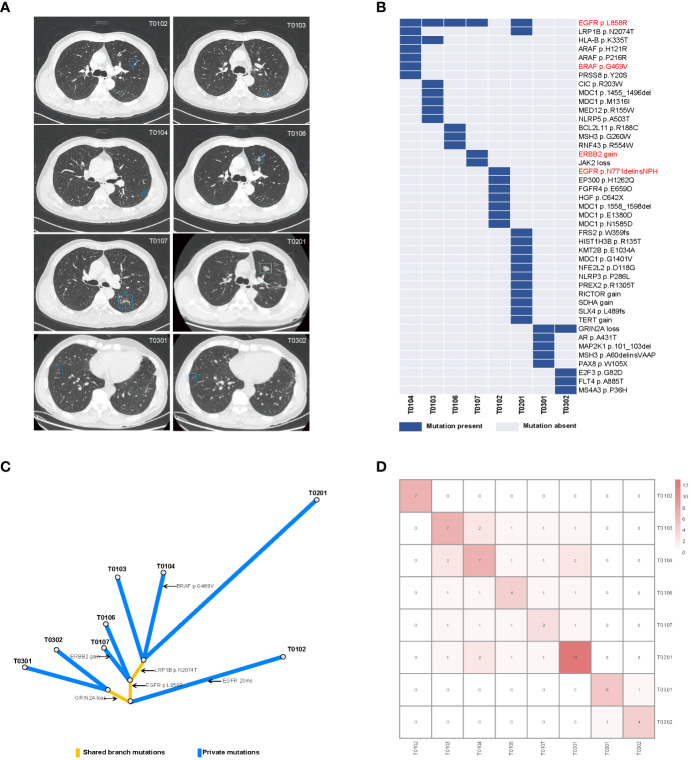
Radiological features and mutational characteristics of GGOs. **(A)** CT images of the eight nodules recognized by the artificial intelligence mode; **(B)** heatmap showing the presence (blue) or absence (gray) of somatic mutations in each tumor; the mutations highlighted in red are clinically actionable variants; **(C)** phylogenetic trees indicating the clonal structure of the tumors sequenced from this patient; **(D)** the number of shared somatic mutations between tumor pairs; CT, computed tomography.

A 55-year-old man former smoker with a family history of lung cancer was admitted to hospital in December 2019 with diffusive multiple nodules in bilateral pulmonary. In total, 109 nodules were distinguished by an artificial intelligence model, including 74 nodules with a diameter of at least 4 mm. Most of the nodules were pure GGOs (pGGOs) (91.7%), and the rest exhibited very little deterioration. Since this patient had severe gastroesophageal reflux disease (GERD) and was treated accordingly, partial resolution of the lesions was noted. We surgically treated malignant lesions at high risk in the left upper and lower lobes with wedge resection via video-assisted thoracoscopic surgery. Histopathological review of the five surgical tumors (T0102, T0103, T0104, T0106, and T0107) revealed no lymph node metastasis. After one year of follow-up, a rapidly enlarging tumor (T0201) in the left upper lobe was observed and surgically resected. Given that multiple tumors harbored *EGFR* mutations and evidence of metastasis, the patient received osimertinib treatment, and the number of lesions (≥ 4 mm) decreased from 65 to 42 in one year. In June 2022, the patient underwent surgical resection of two enlarged residual tumors (T0301, T0302) in the right lower lobe that had been planned for resection at the initial surgery. Four continuous blood samples were collected before and after the first and third surgeries (B01-B04), and imaging was performed during treatment monitoring. The tumor and plasma samples collected and the clinical course are summarized in **Figure 1**. In addition, the detailed clinicopathological and radiological characteristics of all eight surgical specimens are summarized in **Figure 2A** and **Supplementary Table 1**.

**Figure 3:**
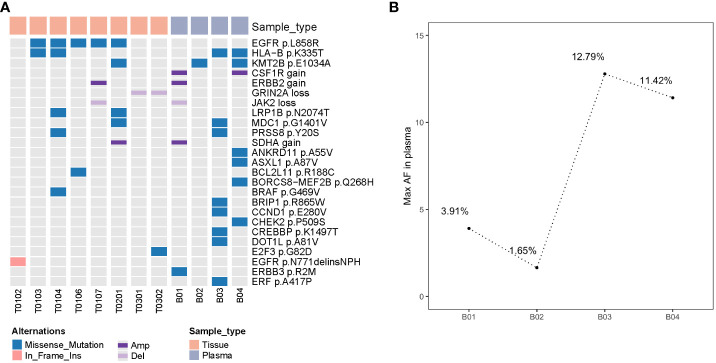
Detection of somatic mutations in four plasma and eight tissue samples from this patient **(A)** molecular mutational profiling of tissue DNA and plasma-derived ctDNA (top 25 mutations); **(B)** the maximal allele fraction of ctDNAs in 4 blood sample; ctDNA mutation burden changes were correlated with treatment response; ctDNA, circulating tumor DNA; Max AF, maximal allele fraction.

We performed deep next-generation sequencing (NGS) of tissue and plasma samples to detect somatic mutations and germline alterations via an AcornMed panel with 808 cancer-associated genes (**Supplementary Methods**, **Supplementary Table 2**) and achieved a mutation allele frequency (AF) of at least 1% for tumor tissue DNA and 0.5% for ctDNA. A plasma sample with at least one variant detected was defined as ctDNA positive. The germline *SPINK1* c.194 + 2T>C was detected. Interestingly, *EGFR* p.L858R was detected in five tumors (T0103, T0104, T0106, T0107, and T0201), which were all diagnosed as stage IA and different pathological phases of AAH, MIA and IAC (**Figures 2A, B**, **Supplementary Table 1**). A phylogenetic tree and shared mutation analysis showed limited relatedness between tumors (**Figures 2C, D**). Based on these two shared mutations, clonal relationships between T0104 and T0103 and between T0104 and T0201 were established, indicating the presence of metastases. Therefore, the patient was diagnosed with both MPLC and IM. Mutations from multiple tissue tumors were detected in B01 and B03 (**Figure 3A**). Notably, mutations in T0103 and T0104 from the first surgery were detected in B03 and B04 (**Figure 3A**). As expected, the maximal allele fraction (Max AF) of ctDNA decreased after the postoperative period (B01, 3.91% vs. B02, 1.65%; B03, 12.79% vs. B04, 11.42%), and the mutation burden (Max AF) of ctDNA increased with disease progression (B02, 1.65% vs. B03, 12.79%) (**Figure 3B**). Currently, the patient is being followed-up without evidence of disease relapse, and he has achieved a recurrence-free survival of 48 months.

## Discussion

We describe a rare case of lung cancer with multiple diffuse GGOs involving the *SPINK1* c.194 + 2T>C germline mutation. The germline variant of *SPINK1* is the most common mutation in Chinese patients with chronic pancreatitis and is closely related to the occurrence of chronic pancreatitis and increased risk of pancreatic cancer (23, 24), but few reports on this topic have been published for lung cancer (25). One patient with the *SPINK1* c.194 + 2T>C germline mutation developed multiple primary tumors, including lung cancer, suggesting that *SPINK1* germline mutations may contribute to the development of multiple primary tumors (26). The patient in our report also had a family history of lung cancer, and this was the first report that the germline *SPINK1* c.194 + 2T>C germline mutation could be associated with multiple GGOs. GERD is a risk factor for NSCLC (27–30). Shivantha Amarnath et al. hypothesized that chronic microaspirations in GERD patients result in a chronic inflammatory state within the lung parenchyma, triggering specific proliferative signaling pathways that may lead to malignant transformation (27). After the patient received treatment for GERD, the number of lesions substantially decreased from 109 to 75, indicating that GERD may have been another factor that caused the patient to develop multiple GGOs with chronic inflammatory or precancerous conditions.

This is an extremely rare lung cancer with the same germline characteristics that exhibits “four phases” of lung adenocarcinoma (31), from AAH to IAC, and presents imaging features favoring MPLC, as no signs of metastasis were found. We investigated the clonal relationship of multiple GGOs, which may have aided in the diagnosis of the tumors. We found that the most common mutations in early GGOs like MIA were *EGFR* and *BRAF* variations and *ERBB2* amplification and that patients with IAC had a greater mutational burden than did patients with other adenocarcinoma subtypes, suggesting that these mutations are key genomic events before the acquisition of invasiveness. There was strong intertumor heterogeneity between tumors, distinct mutational landscapes and weak correlations. Multiple GGOs are generally considered MPLCs. Interestingly, in two tumor pairs, two shared mutations each were detected, which were more similar to inheritance from a common ancestor. Among them, two pGGOs and one AAH were found, which suggested that metastasis can occur in pGGOs and AAHs (2). Combining histologic findings and molecular features, the diagnosis of this patient favored the coexistence of both MPLC and IM, suggesting that targeted sequencing can be used to improve the diagnostic process and that multiple GGOs may represent IM.

Varying degrees of progression have been reported for unresected GGOs, and close postoperative monitoring is essential for these patients (12). ctDNA has been reported as being used to track tumor burden and monitor MRD (19, 32, 33), but little is known about multiple tumors. Among the continuous ctDNA samples of this patient during treatment, few mutations were detected, but no *EGFR* mutations were detected, which may be due to the lower mutational burden and the nonshedding vulnerability of ctDNA in early-stage NSCLC (18), the indolent course of GGOs (34), the progression of other lesions that do not carry *EGFR* mutations and the timing of ctDNA testing. Therefore, sequential monitoring of MRD may improve the sensitivity of patients to adjuvant therapy (35). However, we cannot rule out the absence of detectable *EGFR* for B3 and B4 as a reason for the use of osimertinib. Our results showed that ctDNA detection may not only be able to overcome the heterogeneity of tumors but also more comprehensively reflect the disease state of patients. Before the third operation, we found an increase in the tumor mutation load of ctDNA, which was consistent with the increase observed by imaging some lesions, suggesting that ctDNA may be able to reflect disease progression earlier than imaging and prompt the appropriate timing of surgery. Unfortunately, due to the COVID-19 pandemic, this patient did not undergo ctDNA testing for one year after osimertinib treatment until progressive tumors were observed by imaging. To our knowledge, this is the first case report of molecular residue exploration based on ctDNA in a patient with multifocal lung cancer.

Some GGOs have a poor prognosis, and diffuse multifocal GGOs usually confuse us with the controversial management of unresected GGOs after primary tumor resection. GGOs are insensitive to chemotherapy and anti-PD-1/PD-L1-based monotherapy or combination therapy (36, 37). *EGFR*-TKIs may be a treatment option for multiple GGOs with *EGFR* mutations (38, 39). Considering that *EGFR* was detected in many tumors and that two pairs of tumors were more likely to be metastatic, the patient was subjected to osimertinib treatment after the second operation and maintained stable disease until one year later. **Figure 1**, lower part of the imaging monitoring, illustrates three scenarios of lesion size changes before and after osimertinib administration. These results suggested that targeted therapy is effective for multiple GGOs but is limited (40) because tumors harboring targeted mutations may not be able to effectively represent other tumors without target mutation or NGS detection, and the discrepancy rate of driver mutations in MPLCs is relatively high (41–45). In this case, we also detected *BRAF* mutation and *ERBB2* amplification, which may have affected the efficacy of osimertinib. Therefore, the existence of multicentric tumors with different molecular landscapes in the same patient, especially at the early stage of disease, may challenge treatment decisions. Despite the heterogeneity of the different tumors in this patient, targeted therapies combined with surgery constituted a reasonable alternative strategy, which needs to be verified in a larger sample. At the time of this report, this patient had stable disease without progression and was under close surveillance with continued treatment with osimertinib, indicating the rationality of *EGFR*-TKI treatment. This rare case involves several unexplained clinical realities, and there is currently no consensus treatment. The diagnostic and treatment criteria are not yet well understood, and this case may provide some direction and evidence.

## Conclusions

In summary, encountering such a large number of GGOs in a patient is rare. Genomic profiling may aid in the determination of the exact clonal origin of patients with multifocal lung cancer, possibly helping to rationalize treatment and improve patient prognosis. We believe that resection of dominant GGOs combined with appropriate adjuvant targeted therapy when metastasis occurs and close follow-up of the remaining nodules or monitoring disease progression by ctDNA may be a safe and effective strategy for the treatment of multiple GGOs.

## Data availability statement

The datasets presented in this study can be found in online repositories. The name of the repository and accession number can be found in the article/Supplementary Material. Accession of the submission is HRA006448 (https://ngdc.cncb.ac.cn/search/?dbId=hra&q=HRA006448.

## Ethics statement

The studies involving humans were approved by Medical Ethics Committee of Beijing Haidian Hospital (No. 2020-041). The studies were conducted in accordance with the local legislation and institutional requirements. The participants provided their written informed consent to participate in this study. Written informed consent was obtained from the individual(s) for the publication of any potentially identifiable images or data included in this article.

## Author contributions

YY: Data curation, Supervision, Writing – original draft. GP: Data curation, Supervision, Writing – review & editing. ML: Formal analysis, Visualization, Writing – original draft. XXM: Formal analysis, Visualization, Writing – original draft. SW: Data curation, Supervision, Writing – review & editing. XJM: Data curation, Writing – original draft. SM: Data curation, Supervision, Writing – review & editing. JQ: Writing – review & editing. HW: Writing – review & editing. JL: Writing – review & editing. YH: Writing – review & editing, Conceptualization, Data curation, Project administration, Supervision.
