# Supplementary material for: Case report: Targeted sequencing facilitates the diagnosis and management of rare multifocal pure ground-glass opacities with intrapulmonary metastasis
Source: Front Oncol. 2024 Jan 23;13:1276095. doi: 10.3389/fonc.2023.1276095 (PMC10846301; doi:10.3389/fonc.2023.1276095)
Supplement: Supplementary file 1 [file DataSheet_1.docx]

***Supplementary Material***

1. **Supplementary Table 1**
2. **Supplementary Table 2**
3. **Supplementary Methods**

**Supplementary Table**

Supplementary Table 1. The detailed clinicopathological characteristics of the tumors of the patient.

| Tumor | Therapy | Location | Radiological feature | Size (mm) | Histology | pathological stage | *EGFR* VAF (%) |
| --- | --- | --- | --- | --- | --- | --- | --- |
| T0102 | VATS | LUL | pGGO | 9.9*5.9 | IAC | ⅠA1(T1aN0M0) | 5.92 |
| T0103 | VATS | LLL | pGGO | 3.9*3.5 | AAH | AAH | 0.32 |
| T0104 | VATS | LLL | pGGO | 3*3 | MIA | ⅠA1 (T1a(mi)N0M0) | 0.34 |
| T0106 | VATS | LUL | mGGO | 14.3*9.5 | MIA | ⅠA1 (T1a(mi)N0M0) | 1.37 |
| T0107 | VATS | LLL | mGGO | 25.1*16.7 | IAC | ⅠA2 (T1bN0M0) | 18.38 |
| T0201 | VATS | LUL | mGGO | 19.0*13.7 | IAC | ⅠA2 (T1bN0M0) | 42.53 |
| T0301 | VATS | RLL | pGGO | 7.9*6.3 | MIA | ⅠA1 (T1a(mi)N0M0) | None |
| T0302 | VATS | RLL | pGGO | 5.7*4.3 | AIS | 0 (TisN0M0) | None |

VATS, video-assisted thoracoscopic; LUL, left upper lobe; LLL, left lower lobe; RLL, right lower lobe; pGGO, pure GGO; mGGO, mixed GGO; AAH, atypical adenocarcinoma hyperplasia; AIS, adenocarcinoma in situ; MIA, minimally invasive adenocarcinoma; IAC, invasive adenocarcinoma; VAF, variant allele frequency.

Supplementary Table 2. List of 808 cancer-associated genes in Acornmed panel

| ABCB1 | ABCC1 | ABCC2 | ABCC3 | ABCC6 | ABCC9 | ABCG2 | ABL1 | ABL2 |
| --- | --- | --- | --- | --- | --- | --- | --- | --- |
| ACTB | ACTG1 | ACVR1 | ACVR1B | ACVR2A | ADAMTS12 | ADGRA2 | ADGRG4 | ADH1C |
| AGO2 | AKT1 | AKT2 | AKT3 | ALDOC | ALK | ALOX12B | AMER1 | AMOT |
| ANK2 | ANKRD11 | APC | APCDD1 | APOB | APOE | AR | ARAF | ARFRP1 |
| ARID1A | ARID1B | ARID2 | ARID5B | ASB18 | ASTN1 | ASTN2 | ASXL1 | ASXL2 |
| ASXL3 | ATIC | ATM | ATP7A | ATR | ATRIP | ATRX | AURKA | AURKB |
| AXIN1 | AXIN2 | AXL | B2M | BABAM1 | BACH1 | BAP1 | BARD1 | BBC3 |
| BCL10 | BCL2 | BCL2L1 | BCL2L11 | BCL2L2 | BCL6 | BCOR | BCORL1 | BCR |
| BIRC3 | BLM | BMPR1A | BORCS8-MEF2B | BRAF | BRCA1 | BRCA2 | BRD4 | BRINP3 |
| BRIP1 | BTG1 | BTK | C14orf177 | C6orf118 | CA10 | CALR | CARD11 | CARM1 |
| CASP7 | CASP8 | CBFB | CBL | CBR1 | CBR3 | CCDC6 | CCND1 | CCND2 |
| CCND3 | CCNE1 | CD274 | CD276 | CD74 | CD79A | CD79B | CDA | CDC42 |
| CDC73 | CDH1 | CDH10 | CDH12 | CDH18 | CDH9 | CDK12 | CDK4 | CDK6 |
| CDK8 | CDKN1A | CDKN1B | CDKN2A | CDKN2B | CDKN2C | CEBPA | CENPA | CFTR |
| CHD2 | CHD4 | CHEK1 | CHEK2 | CHRM2 | CHST3 | CHUK | CIC | CNTNAP2 |
| CNTNAP5 | COL22A1 | CRBN | CREBBP | CRKL | CRLF2 | CSDE1 | CSF1R | CSF3R |
| CSMD3 | CTCF | CTLA4 | CTNNA1 | CTNNA2 | CTNNB1 | CUL3 | CUL4A | CUL4B |
| CXCR4 | CYBA | CYLD | CYP17A1 | CYP2B6 | CYP2C19 | CYP2C8 | CYP2C9 | CYP2D6 |
| CYP2E1 | CYP3A4 | CYSLTR2 | DAXX | DCAF12L1 | DCAF12L2 | DCAF4L2 | DCK | DCLRE1C |
| DCUN1D1 | DDB1 | DDR2 | DICER1 | DIS3 | DMD | DNAJB1 | DNMT1 | DNMT3A |
| DNMT3B | DOT1L | DPYD | DROSHA | DUSP27 | DUSP4 | E2F3 | EED | EGF |
| EGFL7 | EGFR | EIF1AX | EIF4A2 | EIF4E | ELF3 | EML4 | EMSY | ENG |
| EP300 | EPAS1 | EPCAM | EPHA2 | EPHA3 | EPHA5 | EPHA7 | EPHB1 | EPHX1 |
| ERBB2 | ERBB3 | ERBB4 | ERCC1 | ERCC2 | ERCC3 | ERCC4 | ERCC5 | ERF |
| ERG | ERICH3 | ERRFI1 | ESR1 | ESR2 | ETV1 | ETV4 | ETV5 | ETV6 |
| EWSR1 | EZH1 | EZH2 | EZR | F3 | FAM135B | FAM175A | FAM46C | FAM58A |
| FANCA | FANCB | FANCC | FANCD2 | FANCE | FANCF | FANCG | FANCI | FANCL |
| FANCM | FAS | FAT1 | FAT3 | FBN2 | FBXL7 | FBXW7 | FCGR3A | FES |
| FGD1 | FGF10 | FGF12 | FGF14 | FGF19 | FGF23 | FGF3 | FGF4 | FGF6 |
| FGF7 | FGFR1 | FGFR2 | FGFR3 | FGFR4 | FH | FIP1L1 | FLCN | FLT1 |
| FLT3 | FLT4 | FOLR3 | FOXA1 | FOXL2 | FOXO1 | FOXP1 | FRK | FRS2 |
| FRYL | FUBP1 | FYN | G6PC3 | GABRA2 | GABRA6 | GALNT12 | GAPDH | GAST |
| GATA1 | GATA2 | GATA3 | GATA4 | GATA6 | GEN1 | GGH | GID4 | GLI1 |
| GNA11 | GNA13 | GNAQ | GNAS | GOPC | GPR158 | GPS2 | GREM1 | GRID1 |
| GRIK3 | GRIN2A | GRM3 | GRM8 | GSK3B | GSTA1 | GSTM1 | GSTM3 | GSTP1 |
| GSTT1 | H3F3A | H3F3AP4 | H3F3B | H3F3C | HAPLN1 | HCN1 | HDAC9 | HFE |
| HGF | HIST1H1C | HIST1H2BD | HIST1H3A | HIST1H3B | HIST1H3C | HIST1H3D | HIST1H3E | HIST1H3F |
| HIST1H3G | HIST1H3H | HIST1H3I | HIST1H3J | HIST2H3A | HIST2H3C | HIST2H3D | HIST3H3 | HLA-A |
| HLA-B | HNF1A | HOXB13 | HRAS | HSD3B1 | HSP90AA1 | HTR1A | ICOSLG | ID3 |
| IDH1 | IDH2 | IFNGR1 | IFNL3 | IGF1 | IGF1R | IGF2 | IGFL3 | IKBKE |
| IKZF1 | IL10 | IL7R | INHA | INHBA | INPP4A | INPP4B | INPPL1 | INSR |
| INSRR | IQCJ | IRF2 | IRF4 | IRS1 | IRS2 | ITPA | JAK1 | JAK2 |
| JAK3 | JUN | KAT6A | KCNA4 | KCND2 | KCNJ3 | KCNT2 | KDM5A | KDM5C |
| KDM6A | KDR | KEAP1 | KEL | KIF2B | KIF5B | KIT | KLC1 | KLF4 |
| KLHL1 | KLHL6 | KMT2A | KMT2B | KMT2C | KMT2D | KMT5A | KNSTRN | KRAS |
| LATS1 | LATS2 | LEPR | LIG3 | LIG4 | LMO1 | LOC349160 | LPL | LPPR4 |
| LRFN5 | LRIG3 | LRP1B | LRRC4C | LRRIQ3 | LRRK2 | LRRTM4 | LTK | LYN |
| LZTR1 | MAD1L1 | MAGI2 | MALT1 | MAP2K1 | MAP2K2 | MAP2K4 | MAP3K1 | MAP3K13 |
| MAP3K14 | MAP4K3 | MAPK1 | MAPK3 | MAPKAP1 | MAX | MBD4 | MCL1 | MDC1 |
| MDH2 | MDM2 | MDM4 | MED12 | MEF2B | MEN1 | MET | MGA | MITF |
| MKRN3 | MLH1 | MLH3 | MNAT1 | MOCS2 | MPL | MRE11A | MS4A3 | MSH2 |
| MSH3 | MSH6 | MSI1 | MSI2 | MST1 | MST1R | MTHFD1 | MTHFR | MTOR |
| MUTYH | MYB | MYC | MYCL | MYCN | MYD88 | MYOD1 | NAT2 | NAV3 |
| NBN | NCAM1 | NCOA3 | NCOA4 | NCOR1 | NEGR1 | NEIL1 | NEIL3 | NF1 |
| NF2 | NFE2L2 | NFKBIA | NKX2-1 | NKX3-1 | NLRP3 | NLRP5 | NOS3 | NOTCH1 |
| NOTCH2 | NOTCH3 | NOTCH4 | NPM1 | NQO1 | NRAS | NSD1 | NTHL1 | NTM |
| NTRK1 | NTRK2 | NTRK3 | NUF2 | NUP93 | NUTM1 | PAK1 | PAK3 | PAK6 |
| PAK7 | PALB2 | PALLD | PAPPA2 | PARK2 | PARP1 | PARP2 | PARP3 | PARP4 |
| PAX5 | PAX8 | PBRM1 | PCDH10 | PCDH17 | PDCD1 | PDCD1LG2 | PDGFRA | PDGFRB |
| PDHA2 | PDK1 | PDPK1 | PDYN | PDZRN3 | PER1 | PGR | PHOX2B | PIK3C2B |
| PIK3C2G | PIK3C3 | PIK3CA | PIK3CB | PIK3CD | PIK3CG | PIK3R1 | PIK3R2 | PIK3R3 |
| PIM1 | PIP5K1A | PLCG2 | PLK2 | PMAIP1 | PML | PMS1 | PMS2 | PNRC1 |
| POLD1 | POLDIP2 | POLE | POLM | POM121L12 | PPARD | PPARG | PPM1D | PPP2R1A |
| PPP4R2 | PPP6C | PRDM1 | PRDM14 | PREX2 | PRIM2 | PRKACA | PRKAR1A | PRKCI |
| PRKD1 | PRKDC | PRSS1 | PRSS8 | PTCH1 | PTEN | PTP4A1 | PTPN11 | PTPRD |
| PTPRS | PTPRT | PXDNL | QKI | RAB35 | RAC1 | RAC2 | RAD21 | RAD50 |
| RAD51 | RAD51B | RAD51C | RAD51D | RAD52 | RAD54L | RAF1 | RANBP2 | RARA |
| RASA1 | RB1 | RBBP8 | RBM10 | RECQL | RECQL4 | REG3A | REL | RET |
| REV1 | REV3L | RFWD2 | RHBDF2 | RHEB | RHOA | RICTOR | RINT1 | RIT1 |
| RNF43 | ROCK1 | ROS1 | RP1L1 | RPA1 | RPL11 | RPL35A | RPL5 | RPP30 |
| RPS10 | RPS17 | RPS19 | RPS24 | RPS26 | RPS6KA4 | RPS6KB2 | RPS7 | RPTOR |
| RRAGC | RRAS | RRAS2 | RRM1 | RRM2 | RTEL1 | RUNX1 | RUNX1T1 | RXRA |
| RYBP | RYR2 | SALL1 | SDC4 | SDHA | SDHAF2 | SDHB | SDHC | SDHD |
| SESN1 | SESN2 | SESN3 | SETBP1 | SETD2 | SETMAR | SF3B1 | SH2B3 | SH2D1A |
| SHMT1 | SHOC2 | SHQ1 | SLC14A2 | SLC19A1 | SLC22A2 | SLC22A4 | SLC34A2 | SLC45A2 |
| SLC8A1 | SLCO1B1 | SLCO1B3 | SLIT2 | SLIT3 | SLITRK1 | SLITRK2 | SLITRK3 | SLX4 |
| SMAD2 | SMAD3 | SMAD4 | SMARCA4 | SMARCB1 | SMARCD1 | SMO | SMYD3 | SNCAIP |
| SOCS1 | SOS1 | SOX10 | SOX17 | SOX2 | SOX9 | SPEN | SPG7 | SPHKAP |
| SPINK1 | SPOP | SPRED1 | SPTA1 | SRC | SRSF2 | ST6GAL2 | STAG2 | STAT3 |
| STAT4 | STAT5A | STAT5B | STK11 | STK19 | STK40 | STT3A | SUFU | SUZ12 |
| SYK | TAF1 | TAP1 | TAP2 | TBP | TBX3 | TCEB1 | TCF3 | TCF7L2 |
| TDG | TEK | TEKT4 | TERC | TERT | TET1 | TET2 | TFE3 | TFEB |
| TG | TGFB1 | TGFBR1 | TGFBR2 | TGFBR3 | TIPARP | TLR4 | TMEM127 | TMPRSS2 |
| TNFAIP3 | TNFRSF14 | TNFRSF17 | TOP1 | TOP2A | TOPBP1 | TP53 | TP53BP1 | TP63 |
| TPM3 | TPMT | TRAF2 | TRAF7 | TRIM58 | TRPC5 | TRRAP | TSC1 | TSC2 |
| TSHR | TSHZ3 | TYK2 | TYMP | TYMS | U2AF1 | UGT1A1 | UGT1A8 | UPF1 |
| USP1 | VEGFA | VHL | VTCN1 | WHSC1 | WHSC1L1 | WISP3 | WRN | WT1 |
| WWTR1 | XIAP | XIRP2 | XPO1 | XRCC1 | XRCC2 | XRCC3 | YAP1 | YES1 |
| ZAN | ZBTB2 | ZFHX3 | ZFHX4 | ZIC1 | ZIC4 | ZIM2 | ZNF217 | ZNF423 |
| ZNF521 | ZNF536 | ZNF703 | ZNF804A | ZNF804B | ZNF831 | ZRSR2 |  |  |

**Supplementary Methods**

**Sample preparation and targeted multigene panel sequencing**

Genomic DNA was isolated from lung nomal and tumor tissue using the Tissue Kit (Qiagen) following the manufacturer’s instructions. ctDNA was isolated from ≥2 ml plasma with the QIAamp Circulating Nucleic Acid kit (Qiagen). The quality and quantity of DNA were assessed using the Agilent 2100 BioAnalyzer (Agilent Technologies) and Qubit dsDNA HS assay kit (Thermo Fisher Scientific). Genomic DNA was sheared to 200~250 bp with a Covaris M220 ultrasonicator (Covaris) before library construction. Indexed NGS libraries were prepared using the KAPA Hyper Library Preparation Kit (Roche KAPABIOSYSTEMS) and enriched via a Twist Custom Panels (Twist Bioscience) targeting 808 cancer-associated genes (collected from the Catalogue of Somatic Mutations in Cancer (COSMIC), The Cancer Genome Atlas (TCGA) and internal databases) and covering more than 2.0 Mb coding genome. Target-enriched libraries were pooled and sequenced on an Illumina NovaSeq 6000 platform (Illumina) with paired 150-bp read length. Mean target sequencing coverage was 1,200× for tumor tissue, 2,500× for ctDNA, and 300× for normal tissue.

**Sequence alignment and variant annotation**

The raw sequencing reads were first subjected to quality control by trimming adaptor sequences and removing the reads with poly-N and low quality preprocessed by FASTP^1^. Then, high-quality reads were aligned to a human reference genome (GRCh37) with Burrows-Wheeler Aligner (BWA)^2^, and duplicate reads by PCR were removed by Picard tools. The subsequent data preprocessing and variant calling were based on the Sentieon Genomics pipeline^3^. Matched genomic DNA from normal lung tissue was used as a control to identify germline DNA. Germline variants were called using Genome Analysis Toolkit (GATK, version 3.8)^4^. The potential P/LP mutations were validated by first-generation sequencing, and pathogenicity was then manually determined by molecular pathologists according to the American College of Medical Genetics standard^5^. Somatic single-nucleotide variants (SNVs) and small insertions or deletions (INDELs) were analyzed using Sentieon Genomics. The recommended parameters were used, including 1) a mutation allele frequency (AF) at least 1% for tumor tissue DNA and 0.5% for ctDNA; 2) ignoring all silent mutations; 3) at least 10 high-quality reads supporting the mutant were observed in any tumor or plasma sample with at least 5 reads on each strand (forward and reverse). SNVs and INDELs were annotated with ANNOVAR^6^. Somatic copy number analysis was performed using PureCN (version 1.19.13)^7^. The driver gene hotspot mutations were defined as described previously^8^. In total, deep sequencing successfully identified 50 somatic mutations in eight tumors and 77 mutations in all 4 plasma samples, with a median of 18.5 mutations per sample.

For cfDNA variants, if the following stringent conditions were met, they were considered to be true somatic mutations: (i) for hotspot mutations, ≥4 high-quality support reads, or for non-hotspots, at least ≥8 support reads; and (ii) clonal hematopoiesis were filtered through deep sequencing of paired normal tissue and white blood cell. The detection limit achieved 0.5% for SNV or small indel detection. A plasma sample with at least one variant detected was defined as ctDNA positive. For each tumor-specific allele inspected in ctDNA at each time point, the wide type (WT) bases and the mutant (MT) bases occurrence were counted to calculate the mutant AF, where AF = MT / (MT + WT). The plasma ctDNA mutation burden was calculated by the maximum mutant AF (Max AF) for all mutations used for detection calling^9,10^.

**Phylogenetic tree construction**

To generate the phylogenetic trees from somatic variants, we leveraged a published computational method named LICHeE (Lineage Inference for Cancer Heterogeneity and Evolution)^11^ to reconstruct multi-sample cell lineage trees and infer the subclonal composition of each sample using variant allele frequencies of somatic SNV. The lineage tree of the somatic SNV clusters was built based on the constraint network^11,12.^

**Reference**

1. Chen S, Zhou Y, Chen Y, Gu J. fastp: an ultra-fast all-in-one FASTQ preprocessor. *Bioinformatics* (2018) 34(17): i884-i90.doi:10.1093/bioinformatics/bty560

2. Kimura K, Koike A. Ultrafast SNP analysis using the Burrows-Wheeler transform of short-read data. *Bioinformatics* (2015) 31(10): 1577-83.doi:10.1093/bioinformatics/btv024

3. Kendig KI, Baheti S, Bockol MA, Drucker TM, Hart SN, Heldenbrand JR, et al. Sentieon DNASeq Variant Calling Workflow Demonstrates Strong Computational Performance and Accuracy. *Front Genet* (2019) 10: 736.doi:10.3389/fgene.2019.00736

4. McKenna A, Hanna M, Banks E, Sivachenko A, Cibulskis K, Kernytsky A, et al. The Genome Analysis Toolkit: a MapReduce framework for analyzing next-generation DNA sequencing data. *Genome Res* (2010) 20(9): 1297-303.doi:10.1101/gr.107524.110

5. Richards S, Aziz N, Bale S, Bick D, Das S, Gastier-Foster J, et al. Standards and guidelines for the interpretation of sequence variants: a joint consensus recommendation of the American College of Medical Genetics and Genomics and the Association for Molecular Pathology. *Genet Med* (2015) 17(5): 405-24.doi:10.1038/gim.2015.30

6. Wang K, Li M, Hakonarson H. ANNOVAR: functional annotation of genetic variants from high-throughput sequencing data. *Nucleic Acids Res* (2010) 38(16): e164.doi:10.1093/nar/gkq603

7. Riester M, Singh AP, Brannon AR, Yu K, Campbell CD, Chiang DY, et al. PureCN: copy number calling and SNV classification using targeted short read sequencing. *Source Code Biol Med* (2016) 11: 13.doi:10.1186/s13029-016-0060-z

8. Pei G, Li M, Min X, Liu Q, Li D, Yang Y, et al. Molecular Identification and Genetic Characterization of Early-Stage Multiple Primary Lung Cancer by Large-Panel Next-Generation Sequencing Analysis. *Front Oncol* (2021) 11: 653988.doi:10.3389/fonc.2021.653988

9. Chen K, Zhao H, Shi Y, Yang F, Wang LT, Kang G, et al. Perioperative Dynamic Changes in Circulating Tumor DNA in Patients with Lung Cancer (DYNAMIC). *Clin Cancer Res* (2019) 25(23): 7058-67.doi:10.1158/1078-0432.CCR-19-1213

10. Zill OA, Banks KC, Fairclough SR, Mortimer SA, Vowles JV, Mokhtari R, et al. The Landscape of Actionable Genomic Alterations in Cell-Free Circulating Tumor DNA from 21,807 Advanced Cancer Patients. *Clin Cancer Res* (2018) 24(15): 3528-38.doi:10.1158/1078-0432.CCR-17-3837

11. Popic V, Salari R, Hajirasouliha I, Kashef-Haghighi D, West RB, Batzoglou S. Fast and scalable inference of multi-sample cancer lineages. *Genome Biol* (2015) 16(1): 91.doi:10.1186/s13059-015-0647-8

12. Ricketts C, Popic V, Toosi H, Hajirasouliha I. Using LICHeE and BAMSE for Reconstructing Cancer Phylogenetic Trees. *Curr Protoc Bioinformatics* (2018) 62(1): e49.doi:10.1002/cpbi.49
